# Prenatal Diagnosis of Reno-Urinary Malformations in a Tertiary Center of Republic of Moldavia

**DOI:** 10.3390/diagnostics14192243

**Published:** 2024-10-08

**Authors:** Victor Roller, Angela Ciuntu, Elena Țarcă, Nicolae Sebastian Ionescu, Teodora-Simina Drăgoiu, Jana Bernic, Eva Gudumac, Emil Ceban, Ana Mișina, Tatiana Băluțel, Adriana Ignat, Liliana Fuior-Bulhac, Dana Elena Mîndru

**Affiliations:** 1 Discipline of Pediatric Surgery, “Nicolae Testemițanu” State University of Medicine and Pharmacy, MD-2004 Chisinau, Moldova; victor.roller@usmf.md (V.R.); jana.bernic@usmf.md (J.B.); eva.gudumac@usmf.md (E.G.); emil.ceban@usmf.md (E.C.); anna_mishina@mail.ru (A.M.); 2 Department of Obstetrics and Gynaecology, Public Health Institution, Institute “Mother and Child”, MD-2004 Chisinau, Moldova; angela.ciuntu@usmf.md (A.C.); fuior.liliana@novamed.md (L.F.-B.); 3 Departament of Pediatrics, “Nicolae Testemițanu” State University of Medicine and Pharmacy, MD-2004 Chisinau, Moldova; tatianabalutel91@gmail.com (T.B.); cibotari.adriana@gmail.com (A.I.); 4 Department Surgery II, Discipline of Pediatric Surgery and Orthopedics, “Grigore T. Popa” University of Medicine and Pharmacy, 700115 Iași, Romania; 5 Department of Pediatric Surgery and Orthopedics, “Carol Davila” University of Medicine and Pharmacy, 020021 Bucharest, Romania; 6 Romanian Academy of Medical Sciences, 030167 Bucharest, Romania; 7 “Carol Davila” University of Medicine and Pharmacy, 020021 Bucharest, Romania; teodora-simina.ionescu@rez.umfcd.ro; 8 Departament of Pediatrics, “Grigore T. Popa” University of Medicine and Pharmacy, 700115 Iași, Romania; mindru.dana@umfiasi.ro

**Keywords:** children, malformation uropathies, ultrasound of the urinary system, prenatal diagnosis

## Abstract

Malformative uropathy in children is one of the most common pathological conditions, with an incidence of 5–14% in newborns. Recent research shows that even in the current conditions, they are often diagnosed only in the advanced stages, when Chronic Kidney Disease is already affirming. This study’s objective is to identify urinary tract anomalies, including malformative uropathies in the prenatal stage, using imaging techniques, namely ultrasound of the pregnant uterus. Using prenatal ultrasonography of the pregnant uterus and postnatal clinical and paraclinical examination, we prospectively evaluated a cohort of fifty children with pyelectasia. We describe the demographic and pathological characteristics of patients diagnosed with renal–urinary abnormalities, as well as their postnatal management. A prenatal diagnosis made during the first 15 to 22 weeks of pregnancy enables the evaluation of early malformative uropathies and the determination of the best time to operate in order to minimize complications. When prenatal ultrasonography, fetal karyotype, tissue sample, and embryonic appendages work together, problems may be partially or entirely revealed by these methods due to mistakes made in imaging examinations. In the case of a pregnancy with an antenatal malformation detected, it is necessary for the delivery to take place in a clinic that can provide favorable services for the survival and investigation of the child born with malformative abnormalities.

## 1. Introduction

Malformative uropathy in children is one of the most common pathological conditions, with an incidence of 5–14% in newborns [1]. Recent research shows that, even in the current conditions, they are often diagnosed only in the advanced stages when Chronic Kidney Disease is already affirming. In asymptomatic patients, the diagnosis is complicated, presenting a series of difficulties related to their clinical–evolutionary peculiarities. Several pathophysiological mechanisms are responsible for the functional and organic structural changes in the renal parenchyma. Urinary tract dilation, especially of the pelvis, is a true marker of urinary tract obstruction with urodynamic disorder [2].

Pyelectasis is an extension of the pelvis that impedes the flow of urine. It is considered by many authors as the result of functional or organic changes in the pyeloureteral segment, accompanied by dilation of the pelvis with subsequent changes in urodynamics and requiring instrumental investigations and long-term clinical–paraclinical evaluation [3]. Studies show that several pathophysiological and bacteriological mechanisms are responsible for structural, functional, and organic changes in the renal parenchyma [1,4]. Pyelectasis is recorded in 1–2% of cases, from all malformations in children [5]. Reno-urinary malformations cause particular difficulties both in diagnosis and in solving them, being a real steppingstone in surgery and pediatric urology. In young adulthood, these patients may need renal replacement treatment even if their child’s kidney function is normal. They have a fourfold increased risk of developing end-stage renal disease. [6]. To rule out a possible worsening of the condition, unilateral pyelectasia requires a regular ultrasound examination even after 28 weeks of gestation [7]. A vast majority of authors indicate that severe bilateral hydronephrosis is associated with an increased risk of unsatisfactory results, and for newborns and infants, pyelectasia is considered a borderline condition [8]. The most frequent causes of pyelectasia in this group of patients are pathological pregnancy, which increases the chance of an early birth; the mother’s illnesses; and the newborn’s physiological immaturity [1,9,10]. It is known that the functional ability of the pelvic–ureteral segment of the newborn begins to function normally between 3 and 6 months of age, which explains the transient changes in the kidneys during this period of childhood [6]. Other causes that are associated with the appearance of pyelectasia are of organic origin. However, the exact causes contributing to the development of malformations are not fully known.

The main objective of the study is to identify the types of urinary tract anomalies in children, including malformation uropathies in the prenatal stage, using imaging techniques, namely ultrasound of the pregnant uterus and correlation with postnatal exams, in a prospective study from an Eastern European country. The secondary objectives of the study are the postnatal management of detected anomalies and the identification of anomalies associated with reno-urinary malformations.

## 2. Materials and Methods

### 2.1. Study Population

We prospectively studied 108 pregnancies monitored for suspected malformation of the urinary tract, detected in the antenatal period by ultrasonography of the pregnant uterus, and followed up postnatally, both clinically and paraclinically, between 2014 and 2020. The pregnant mothers were followed by ultrasound at the Public Health Institution, Institute “Mother and Child” Chisinau, Moldova, by two doctors with competence in ultrasonography, authors of this article. After birth, the patients were hospitalized in the pediatric urology clinic of the “Natalia Gheorghiu” National Scientific and Practical Center for Pediatric Surgery, Institute of Mother and Child, Chișinău, Republic of Moldavia. The research project was approved by the Research Ethics Committee of “Nicolae Testemitanu” State University of Medicine and Pharmacy (Approval No. 69 of 30 June 2020). A descriptive statistical process was performed using the standard statistical package JASP Team (2022) (JASP (Version 0.16.4), University of Amsterdam, The Netherlands, https://jasp-stats.org/ (accessed on 14 November 2022).

### 2.2. Inclusion and Exclusion Criteria

All the neonates with reno-urinary tract abnormalities detected antenatally and followed after birth in our pediatric surgery unit were included in the study. The exclusion criteria were a length of hospitalization of under 2 days (1 neonate died in the first two days of life) and transfer for treatment in another hospital (1 neonate with bladder exstrophy and 1 with posterior urethral valves).

### 2.3. Data Collection

The study included the assessment of data related to the health status of the mother and child, risk factors, degree of prematurity (if present), the type of reno-urinary malformation, and the associated anomalies, the latter being then divided into systems.

The antenatal examination by ultrasonography was completed clinically and paraclinically after birth by anamnestic data, clinical examination, and laboratory data (urodynamic tests, nitrogen retention tests (urea, creatinine), bacteriological exploration, and laboratory) and confirmed by ultrasound; intravenous urography; micturition cystourethrography; renal scintigraphy; or computed tomography with angiography, heart Doppler, and encephalography.

### 2.4. Equipment and Procedures

Prenatal ultrasonography (USG) scanning was performed by specialists in fetal ultrasonography. All postnatal USG scanning procedures were performed by the imagist experienced in pediatric urology, using a TOSHIBA Aplio 400 device and a 5 MHz convex and 8 MHz linear probe. The anteroposterior diameter (APD) of the renal pelvis was measured in the transverse plane. The kidneys were evaluated macroscopically for shape, size, location, parenchymal thickness, echogenicity, cysts, and calyceal dilatation. Dilated ureters and bladder diameter and wall thickness were also evaluated. During the ultrasonographic examination, we were able to detect prenatally only abnormalities related to the kidneys and pelvis, namely their dilatation. The other abnormalities developed in the bladder and ureters were determined postnatally by other investigative methods, such as cystoscopy, cystography, and intravenous urography, which are not able to determine wall thickness or anteroposterior diameters.

Voiding cystourethrography (VCUG) and scintigraphy were performed in cases with unilateral or bilateral APD > 15 mm, ureteral dilatation, febrile urinary tract infection during follow-up, pelvic dilatation persisting for more than 12 months, and/or increasing dilatation. Computed tomography was performed in children for whom elevated levels of urea and creatinine did not allow contrast urography to be performed.

### 2.5. Definitions

Hydronephrosis, pyelectasia, polycystic kidney, posterior urethral valve (PUV), ureteropelvic junction obstruction (UPJO), vesicoureteral reflux (VUR), bladder diverticulum, and ureteral hypoplasia diagnosed in the study subjects were classified as congenital anomalies of the kidney and urinary tract (CAKUT).

Pyelectasia is the distension of the renal pelvis without calyceal distension, defined according to the Society for Fetal Urology as an anteroposterior pelvis diameter (APD) of 4 mm at 16 to 27 weeks of gestation and 7 mm at 28 weeks of gestation. The severity of renal dilation can be categorized based on gestational age-specific criteria. At 16 to 27 weeks of gestation, dilation is categorized as mild (4 to <7 mm), moderate (7 to 10 mm), and severe (>10 mm). Beyond 28 weeks of gestation, dilation is categorized as mild (7 to <9 mm), moderate (9 to 15 mm), and severe (>15 mm) [5,10].

Hydronephrosis is differentiated from pyelectasis by larger measurements of the fluid-filled space. Postnatal hydronephrosis was defined according to APD measurement (7–9 mm, mild; 9–15 mm, moderate; and >15 mm, severe). Resolution was defined as an APD < 7 mm at two consecutive USG imaging sessions, and normal renal parenchyma, calyces, ureters, and bladder [11].

VUR was staged according to the International Reflux Study Committee Classification. UPJO was defined as renal pelvis dilatation exceeding 10–15 mm, in the presence of calyceal dilatation and the absence of dilatation of the ureters.

### 2.6. Follow-Up

Subjects diagnosed with reno-urinary abnormalities were followed for 36 months, and during this time, USG, bacteriological examination of urine, blood pressure measurements, and growth measurements were performed every three months. The character of the obstruction, the level, and the calyx/parenchyma ratio were concretized. Dynamic diuretic scintigraphy allowed us to assess the level of obstruction and the condition of the pelvis, and if it is larger than 20 mm, then surgery is performed.

**Figure 1:**
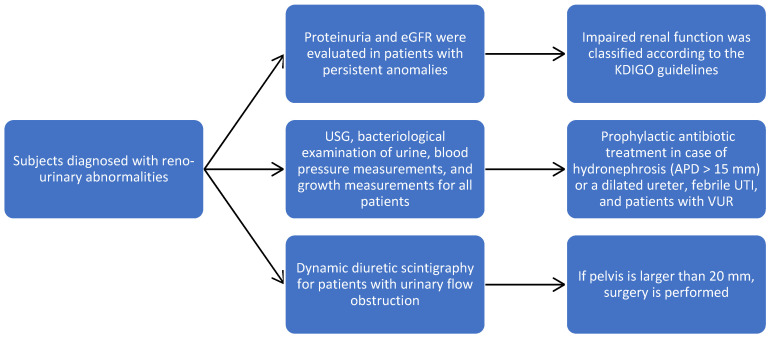
Study flowchart to our patient population.

Proteinuria and the estimated glomerular filtration rate (eGFR) were evaluated in patients with persistent anomalies; eGFR was calculated using the Schwartz formula, and impaired renal function was classified according to the KDIGO guidelines. Prophylactic antibiotic treatment was applied in the case of severe unilateral or bilateral hydronephrosis (APD > 15 mm) or a dilated ureter, febrile UTI, and patients with VUR (Figure 1). The surgical techniques were adapted according to the detected pathology.

### 2.7. Statistical Analysis

The clinical–statistical study included the assessment of the maternal context, data related to sex and degree of prematurity, risk factors, the type of reno-urinary malformation, and the associated anomalies, the latter being then divided into systems. The results were obtained by studying the observation sheets, the primary evidence, and the diagnostic and surgical protocols. A descriptive statistical process was performed using the standard statistical package JASP Team (2022) (JASP (Version 0.16.4), University of Amsterdam, The Netherlands, https://jasp-stats.org/ (accessed on 14 November 2022).

## 3. Results

Out of 108 pregnancies monitored for suspected malformation of the urinary tract in the 2014–2020 period, only 52 fetuses were confirmed with this type of anomaly, and 50 were subsequently monitored after birth in our department; therefore, we calculated a misdiagnosis rate of 51.8%. In the group of 50 patients studied, the distribution in the area of origin indicated the prevalence of patients in the rural sector, 32 (64%); an approximately equal ratio of gender affectation was noted, with a slight prevalence of females, *n* = 28 (56%). This study shows that 36 (71%) of the children come from the first or second pregnancy, 14 (27%) were born prematurely, and 7 (13%) had low birth weight. The age at which prenatal diseases were most commonly diagnosed was 27 weeks of pregnancy.

### 3.1. Outcomes of Postnatal Examination

Of the total number of fetuses examined ultrasonographically in the prenatal period, malformation uropathies (obstructive, refluxing megaureter hydronephrosis, uni- or bilateral hydronephrosis, unilateral multicystic renal dysplasia, etc.) detected at 17–18 weeks of gestation, and then at 20–22, 25–27, and 34–36 weeks of gestation, were confirmed postnatally with urinary tract abnormalities in 98% of cases. During the fetal ultrasound examination, hydronephrosis was identified in 23 cases (68.00%) Stenosis of the pyeloureteral junction was the cause of the development of hydronephrosis in approximately half of the cases, 21 (41.18%). The diagnosis of severe hydronephrosis was established in 6 (17,64%) cases, medium hydronephrosis in 9 (23.53%), and mild in 19 (55,88%) cases. The incidence and characteristics of congenital anomalies of the kidneys and urinary tract in our population are presented in Table 1. In nine children (11.76%), malformations of the urinary system were associated with malformations of other systems, namely esophageal atresia or anorectal malformations. When analyzing the results of our study, we observe a tendency for the development of reno-urinary malformations predominantly on the right side, 57,6%.

### 3.2. Associated Abnormalities

More than half of the patients included in the present study had associated abnormalities, the most common of which were gastrointestinal anorectal malformations, n = 11 (22%); anomalies of the intestines, n = 8 (16%); and cardiovascular n = 2 (4%) with ventricular septal defect (VSD), atrial septal defect (ASD) in three cases (7%), and patent ductus arteriosus (PDA) in four cases (8%). Several urinary abnormalities were recorded simultaneously in a series of patients. We record this trend in Table 2.

### 3.3. Risk Factors

The analyzed statistical data highlighted the risk factors in malformative uropathies, such as the following: maternal—3 cases (6%); intrauterine infections—2 cases (4%); physical, radiation, alcohol consumption, drugs, and chemotherapy—1 case (2%); multifactorial causes—n = 12 (25%); and unidentified causes—32 (63%). Against the background of these risk factors, births with complicated evolution were expected and were found in 27 cases (54%). The associated complications were recorded in the following way: vicious presentations, n = 9 (18%); tight umbilical cord around the neck, n = 32; (64.8%) prolonged birth, n = 24 (48%); and neonatal resuscitation, n = 15 (30%).

### 3.4. Differential Diagnosis

The differential diagnosis was made for all patients with renal megaureter hydrocalycosis, renal cysts, etc. If on the ultrasound of the urinary system the patient had a dilated ureter, ascending cystography was performed to exclude vesicoureteral reflux and infravesical obstruction, and to determine the condition of the bladder.

### 3.5. Conservative or Surgical Treatment

After birth, every 3 months, monitoring via clinical examination and ultrasound of the urinary system was performed. The character of the obstruction, the level, and the calyx/parenchyma ratio were concretized. Dynamic diuretic scintigraphy allowed us to assess the level of obstruction and the condition of the pelvis, and if it is larger than 20 mm, then surgery is performed. Of all the patients followed, 17 (34.00%) underwent surgery, 29 (58%) were dynamically monitored by ultrasonography of the urinary system once every 3 and 6 months, and 8 (8%) were treated with non-invasive methods (urinary antiseptics, antibacterial preparations, and decompression of the urinary bladder by applying the Foley catheter). Of the group of children undergoing surgery, 12 (70.59%) were boys. Intraoperative ureteral hypoplasia was detected in nine (17.65%), and in a similar percentage, multicystic kidney was also diagnosed. Bladder diverticula were identified only in three patients (5.88%).

#### 3.5.1. Clinical Case 1

**Figure 2:**
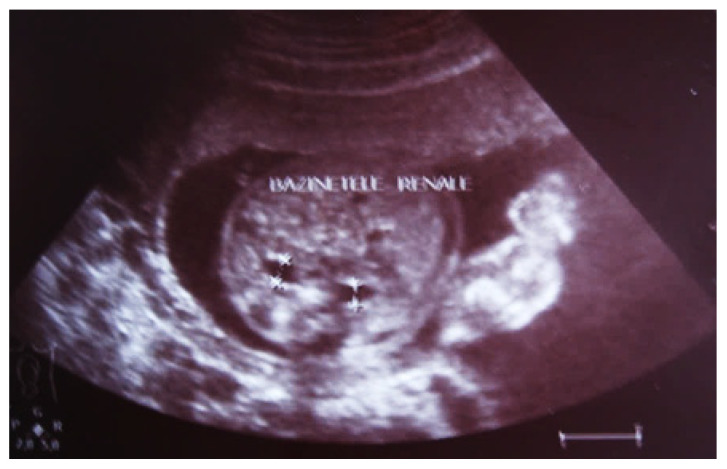
Intrauterine ultrasound at the 21st week of pregnancy. Ultrasonography—the fetus corresponds to the age of 20–21 weeks. Bilateral renal pyelectasia.

**Figure 3:**
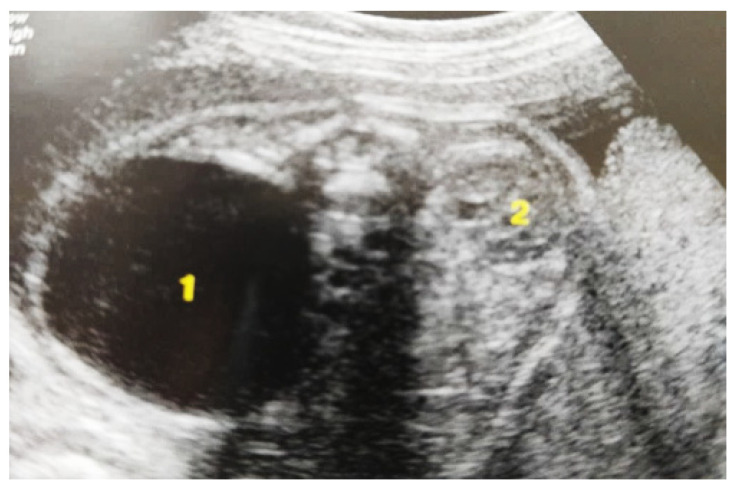
Ultrasound at the 20th week of gestation. Hydronephrosis on the right. (1) Dilated pelvis on the right. (2) Uninjured renal parenchyma on the left.

**Figure 4:**
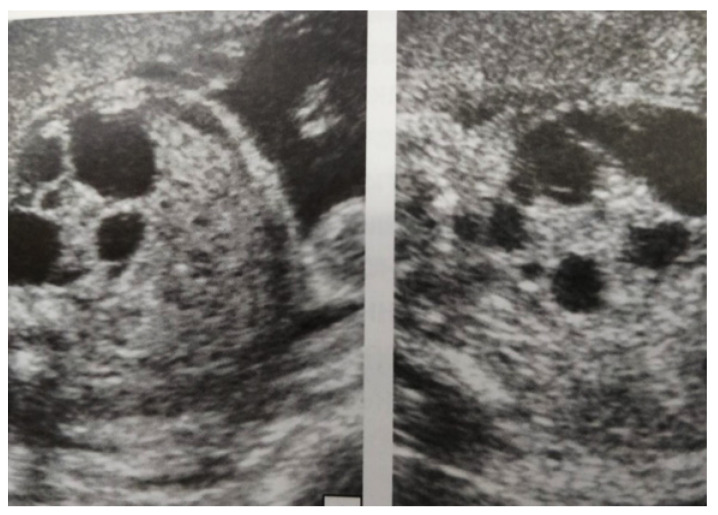
Ultrasound at 31st week of gestation. Bilateral hydrocalconephrosis.

**Figure 5:**
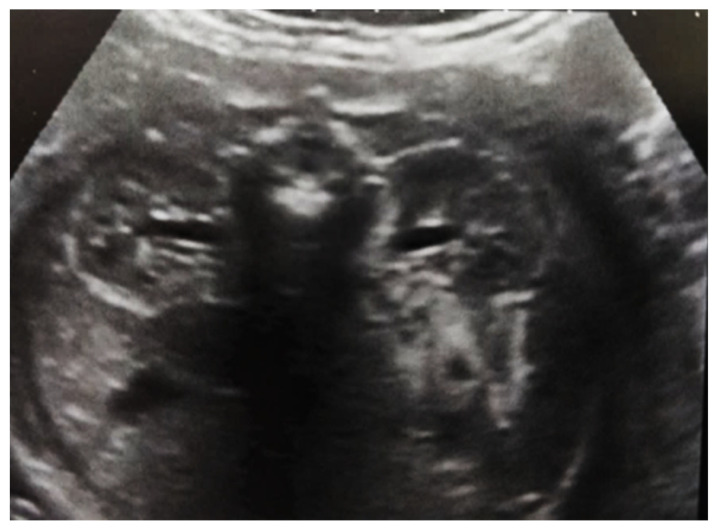
Ultrasound at 32 weeks of gestation. Bilateral pyelectasis.

**Figure 6:**
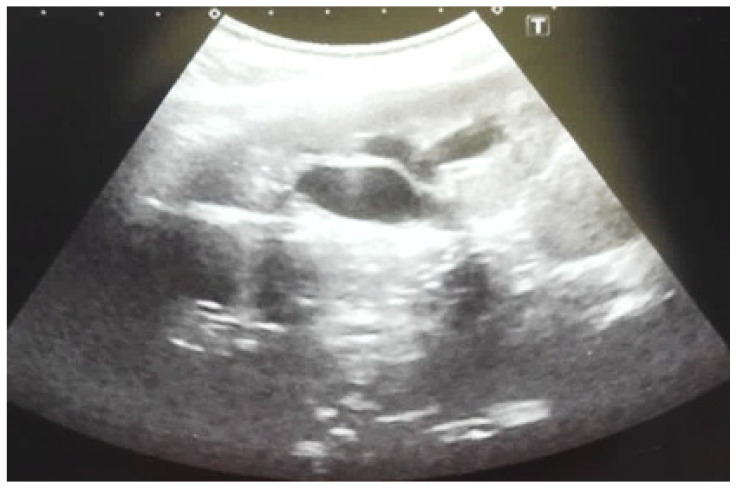
Ultrasound of the postnatal urinary system 10 days after birth. Hydronephrosis on the right.

**Figure 7:**
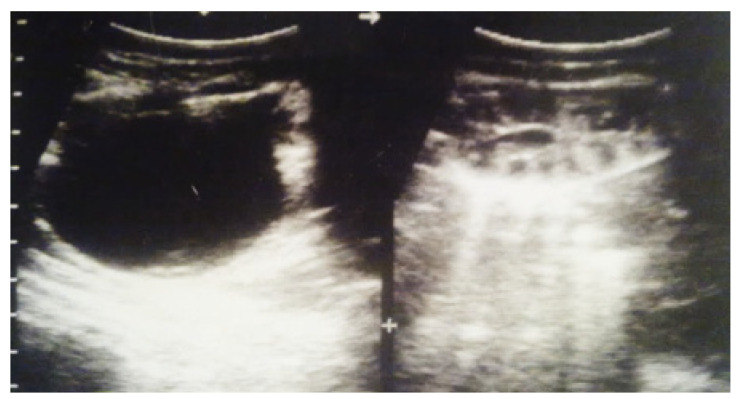
Postnatal urinary ultrasound. Hydronephrosis on the right (newborn 10 days of age).

**Figure 8:**
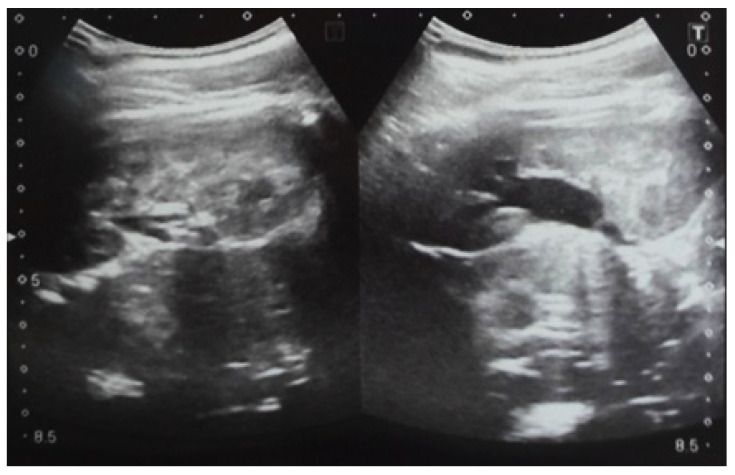
Postnatal urinary ultrasound. Hydronephrosis on the left.

**Figure 9:**
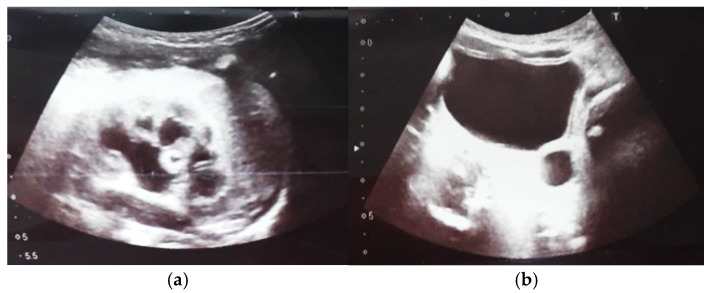
Ultrasound of the urinary system in a 12-day-old newborn. Hydrocalconephrosis on the left (**a**) and ureteral hydronephrosis on the right (**b**).

Our experience with congenital malformations of the urinary system refers to the following clinical case (Figure 2, Figure 3, Figure 4, Figure 5, Figure 6, Figure 7, Figure 8 and Figure 9). In the first case, during pregnancy, the 21-week ultrasound determined bilateral renal pyelectasia. The fetus had hydronephrosis on the right; at 31 weeks, it had bilateral hydrocalconephrosis; and at 32 weeks, bilateral pyelectasia. It suffered premature birth at 36 weeks, with complicated anomalies of the forces of contraction, prolonged birth, and birth weight of 2200 g. Postnatal ultrasonography was supplemented by intravenous urography, and bilateral hydronephrosis was determined. Hydrocalconephrosis on the left was discovered. The complete diagnosis was established: congenital renal malformation; bilateral pyelectasia; bilateral hydronephrosis; hydrocalconephrosis on the left; and severe reduced glomerular filtration rate (GFR), GFR > 2SD below mean.

#### 3.5.2. Clinical Case 2

**Figure 10:**
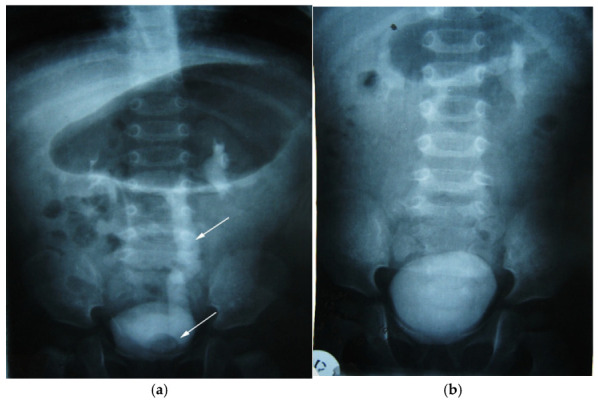
Intravenous urography. Orthotopic left ureterocele, preoperative (**a**) and postoperative after 2 years (**b**). Top arrow—ureter dilated up. Lower arrow—orthotopic ureterocele (simple).

We present another clinical case of a 1-year-old child with an antenatal diagnosis of left hydronephrosis, established at 20 weeks of gestation. Natural birth at 39 weeks, with a circular umbilical cord, birth weight 3310 g. The patient had no symptomatology until one year after birth; the only clinical sign was two-stage emptying of the bladder. The clinical symptoms were that of a reno-urinary condition, to which fever, nausea, vomiting, and abdominal pain were added. Laboratory tests have consistently revealed hyperleukocytosis and increased ESR (Erythrocyte Sedimentation Rate). Ultrasound of the urinary system was the main exploration, which allowed the diagnosis to be established: pyelectasia and left ureterohydronephrosis, with orthotopic ureterocele (simple) on the left. An in-depth examination, supplemented by intravenous urography, confirmed left ureterohydronephrosis of the left kidney with orthotopic (simple) ureterocele (Figure 10a). The child underwent surgical correction according to the clinical protocol. Orthotopic ureterocele excision was performed histopathologically confirmed on the excised fragment. Ureterocystoneoanastomosis with anti-reflux protection on the left. The child was monitored postoperatively according to a scheme used in the pediatric urology clinic (Figure 10b). The study showed no signs of recurrence. The postoperative evolution was devoid of events, and the clinical and paraclinical examination attests to the perfect functionality of the anastomosis and the absence of any sign of recurrence at the two-year follow-up.

## 4. Discussions

Today’s topical issue for pediatricians and nephrologists is congenital pyelectasia, scars in the kidney tissue often accidentally found in children during an ultrasound examination. The given malformations result from errors in morphogenesis. They are manifested by their clinical, morpho-pathological, predictive, and evolutionary diversity and also constitute an alarm signal by themselves and by the associated complications and socio-human and material impact of taking care of these children. The spectrum of outcomes for these patients ranges from no clinical significance to end-stage renal disease [12,13].

This paper includes 50 patients and two consecutive clinical cases that illustrate the need for prenatal diagnosis and early treatment for congenital urinary system abnormalities. After birth, the neonates were followed up by imaging techniques, standard renal scintigraphy, computed tomography, and laboratory and bacteriological examinations. As a result of this study, a series of measures to improve the diagnosis of malformation uropathies by performing antenatal diagnostic screening via ultrasound of the pregnant uterus is required in the Republic of Moldavia. Most studies show that the maternal diet with the use of multivitamin supplements, folic acid, methionine, and minerals (Mg, Zn, and Cu) significantly decreases the incidence of congenital malformation uropathies [11,12,13,14,15].

Urinary tract abnormalities have an incidence of 6–8 per 1000 newborns [1,2]. Studies show that 10% of congenital renal malformations have genetic causes, 10% external and 80% both [16]. At the same time, it is demonstrated that the ultrasound of the internal organs and urinary system performed between the 6th and 11th weeks of pregnancy assesses not only the location and age of the fetus but a large number of associated abnormalities can also be seen, for example, in the brain, heart, intestine, and kidney; in the second and third trimester of pregnancy, sex and structural abnormalities can be determined. Among all the congenital malformations detected antenatally, malformation uropathies of the kidneys have a mesodermal origin and a structure, starting from the mesoderm and endoderm, and it has been demonstrated that the development of the latter is closely related to that of the anus, rectum, and reproductive organs with a frequency of 26 to 28% of the associations [17,18]. In our study, the incidence of malformations associated with urinary tract malformation diagnosed in children (gastrointestinal (38%) and cardiovascular (19%)) was much higher than the results found in the literature. There was an increase in the incidence depending on the period studied, with a maximum between 2016 and 2020.

Urinary tract dilation (UTD), especially of the pelvis, is a true marker of urinary tract obstruction, with urodynamic disorders. In these patients, the organic barrier will inevitably associate with the development of hydronephrosis. Around 1–5% of all pregnancies are affected by fetal UTD abnormalities [3,4]. During pregnancy, the most widely used criterion to select patients requiring post-natal investigation is the third-trimester threshold value for the anteroposterior (AP) renal pelvis diameter of 7 mm [5]. Pelviectasis usually is assessed using the AP measurement criteria; although there is no consensus on this matter, an AP > 4 mm in the second trimester and/or >7 mm in the third trimester is diagnostic [17]. The length of the kidney and adrenal glands, which are compared at a ratio of 2:7, remains constant throughout gestational age. This result may also help identify and assess aberrant growth [18].

According to some studies, fetal distension of the urinary collecting system can spontaneously resolve in about 36–80% of cases after birth, being a simply dynamic and physiologic process [3,7]. Nevertheless, in some situations, renal pelvis dilation can signal the presence of severe urinary tract pathologies, especially in patients with significant hydronephrosis [3]. It is shown that the vast majority of newborns, in whom urinary tract dilation has been detected antenatally, may have a spontaneously favorable postnatal evolution [8].

Conclusive studies are showing that dilation of the renal pelvis of more than 5 mm in the second trimester of pregnancy and more than 8 mm in the third trimester of pregnancy is an argument for complex clinical and paraclinical evaluation of this category of newborns after birth [17,19]. From the multitude of studies, objective data confirm that the dilation of the pelvis up to 10 mm in the third trimester can regress or even disappear after the birth of the child [18,19,20]. Studies show that pelvic dilation in the anteroposterior aspect of more than 10 mm in the fetus in the second trimester of pregnancy is a significant criterion, with a sensitivity of 90% and a specificity of 70%. The dilatation of the pelvis more than 12 mm in the third trimester has a sensitivity of 72% and specificity of 87.3%. The analysis of the data of the specialized literature indicates the need to perform the ultrasonography of the pregnant uterus in three periods: at 10–14 weeks of gestation, at 18–22 weeks, and at 30–32 weeks. Urinary tract malformations diagnosed antenatally have as a causal factor, the obstacle in the flow of urine, creating hypertension in the overlying urinary tract and affecting the renal parenchyma. With the deepening of research on malformation uropathies in newborns and young children diagnosed with malformation uropathies in front of the urologist, the problem of improving antibiotic prophylaxis and medical–surgical techniques and optimizing postoperative evaluation arises [3,21,22,23,24,25].

On a related note, Has et al. report that, among children with a postnatal anteroposterior diameter (AP) > 7 mm and/or kidney, calyceal, ureteral, or bladder pathology, 15% had persistent urinary tract dilatation (UTD) and 32–39% (depending on the method used) had kidney damage [22]. Major postnatal urinary tract ultrasound abnormalities and a congenital anomaly of the kidney and urinary tract (CAKUT) diagnosis were factors associated with an increased risk for permanent kidney damage (odds ratios 8.9, *p* = 0.016; and 14.0, *p* = 0.002, respectively) [22,26,27,28].

*Limitations of the study:* Although Chișinău is the capital of the Republic of Moldova and the most important prenatal diagnosis center in the country, it is possible that some of the expectant mothers have had antenatal ultrasound examinations in other obstetric centers in the country or outside the country, and thus some of the patients with urinary malformations to be lost from our records. Also among the study’s weaknesses is the absence of data on whether the prevalence of reno-urinary malformations has declined since birth as a result of a lack of postnatal diagnosis. Due to the relatively long follow-up period of the patients and the variable availability of the physicians who performed the measurements, we were unable to systematize the data in such a way that we can calculate intraclass correlation coefficients in our study. Due to the small number of patients, the results and conclusions of our study cannot be generalized. To demonstrate the preliminary results and conclusions of the presented study, the increase in the group of studied patients and the comparison with a control group will be the objects of a future study.

As far as we know, this is the largest study carried out in the Republic of Moldova, an Eastern European country, regarding the antenatal diagnosis of reno-urinary malformations, their postnatal management, and their malformation associations. From the data of the specialized literature, corroborated with our experience of 50 cases, we conclude that the reported cases illustrate, once again, the complexity of the etiopathogenic mechanisms of these malformative diseases, which sometimes remain an unknown and challenging interpretation, especially in their asymptomatic forms, and when the clinical manifestations are often characteristic of other diseases of that etiology.

## 5. Conclusions

Based on multicenter studies and randomized trials, the difficulties of antenatal, as well as postnatal, diagnosis for uncharacteristic clinical manifestations, at least for the early stages of the disease, are recognized, and currently, we cannot speak of a “standard” procedure in their diagnosis and treatment. Recent studies show that the etiopathogenesis of reno-urinary congenital malformations results from either a cell differentiation or the involvement of environmental factors (viral and toxic) that can stimulate the genetically predisposed field. An antenatal diagnosis in the 22–28 weeks of gestation allows for the evaluation of early malformation uropathies and selection of the optimal time for surgery that allows the minimum reduction of complications. The collaboration between antenatal ultrasound, fetal karyotype, tissue-sampling embryonic appendages, and enzymatic dosing may reveal abnormalities, but not completely, as a result of errors in imaging investigations. In the case of a pregnancy with an antenatal malformation detected, the delivery must take place in a clinic that can provide favorable services for the survival and investigation of the child born with malformation abnormalities. The increased risk of developing Chronic Kidney Disease in patients with renal–urinary malformations requires close monitoring through periodic, clinical, and paraclinical controls that would allow for the monitoring of the evolution, as well as the complications. The data presented require knowledge of these renal–urinary malformations. Precocity of the surgical act has favorable effects on the prognosis of malformative diseases of the urinary system. Future research will focus on expanding the patient group under study and comparing it to a control group to validate the preliminary findings and conclusions of the study that was presented.

## Figures and Tables

**Table 1:** Incidence and characteristics of congenital anomalies of the kidney and urinary tract.

Total Number of Cases (Percentage)	Prenatal Number of Cases (Percentage)	Postnatal Number of Cases (Percentage)	Right Number of Cases (Percentage)	Left Number of Cases (Percentage)	Bilateral Number of Cases (Percentage)	Male/Female Rate (Percentages)	At Term/ Premature Rate (Percentages)
CAKUT n = 50	50 (100%)	48 (96%)	72 (57.6%)	24 (19.2%)	29 (23.2%)	22/38 (44%/56%)	34/16 (68%/32%)
Pyelectasia 34 (68%)	34 (100%)	34 (100%)	20	6	8	19/15 (55%/45%)	27/17 (79.44%/20.56%)
Hydronephrosis 34 (68%)	23 (68%)	11 (32.35%)	14	8	12	19/15 (55%/45%)	27/17 (79.44%/20.56%)
Severity		Mild 19 (55.88%) Medium 9 (26.47%) Severe 6 (17.64%)					
Megaureter hydronephrosis 9 (17.65%)	2 (22.22%)	7 (77.77%)	4	2	3	3/6 (33.3%/66.7%))	2/7 (22.22%/77.78%)
Pyeloureteral stenosis 21 (41.48%)	0	21 (100%)	15	2	4	11/10 (52.38%/47.61%)	15/6 (71.42%/28.57%)
Vesicoureteral reflux 9 (17.65%)	0	9 (100%)	8	0	1	5/4 (55.6%/44.4%))	6/3 (66.7%/33.3%)
Ureteral hypoplasia 9 (17.65%)	0	9 (100%)	8	0	1	2/7 (22.22%/77.78%)	8/1 (88.9/11.1%)
Multicystic kidney 9 (17.65%)	1 (11.11%)	8 (88.9%)	3	6	NA	6/3 (66.7%/33.3%)	7/2 (77.78%/22.22%)
Diverticula of the bladder 3 (5.88%)	0	3 (100%)	NA	NA	NA	3/0 (100%)	3/0 (100%)

**Table 2:** Concomitant association of several reno-urinary malformations.

	Diverticula of the Bladder (n = 9)	Multicystic Kidney (n = 9)	Ureteral Hypoplasia (n = 9)	Pyeloureteral Stenosis (n = 21)	Megaureter Hydronephrosis (n = 9)	Hydronephrosis (n = 34)			Vesicoureteral Reflux (n = 9)
						Mild	Medium	Severe	
Pyelectasia (n = 34)	1 (2.94%)	3 (8.82%)	6 (17.65%)	21 (61.76%)	6 (17.65%)	14 (41.18%)	8 (23.53%)	6 (17.65%)	6 (17.65%)
Hydronephrosis (n = 34)	1 (2.94%)	1 (11.11%)	-	15 (44.12%)	-	-			-
Megaureter hydronephrosis (n = 9)	-	-	9 (100%)	2 (22.22%)	-	8 (23.53%)			-
Pyeloureteral stenosis (n = 21)	-	1 (11.11%)	2 (9.52%)	-	-	-			3 (33.33%)
Ureteral hypoplasia (n = 9)	-	-	-	2 (9.52%)	9 (100%)	9 (26.47%)			5 (55.56%)
Vesicoureteral reflux (n = 9)	2 (22.22%)	-	-	-	5 (55.56%)	3 (33.33%)	2 (22.22%)	1 (11.11%)	-

## Data Availability

The raw data supporting the conclusions of this article will be made available by the authors on request.
